# Misdiagnosis of uveal melanoma and other intraocular malignancies in enucleated eyes: a 28-year national review

**DOI:** 10.1186/s40942-026-00927-x

**Published:** 2026-08-27

**Authors:** Hans Witzenhausen, Katerina Stripling, Gustav Stålhammar

**Affiliations:** 1 Department of Clinical Neuroscience, Division of Eye and Vision, Karolinska Institutet, Stockholm, Sweden; 2 St. Erik Ophthalmic Pathology Laboratory, St. Erik Eye Hospital, Stockholm, Sweden; 3 Ocular Oncology Service, St. Erik Eye Hospital, Stockholm, Sweden

**Keywords:** Uveal melanoma, Pseudomelanoma, Enucleation, Diagnostic error, Ophthalmic pathology

## Abstract

**Background:**

Intraocular tumors are often diagnosed clinically rather than by biopsy, so a benign lesion can be mistaken for malignancy and lead to enucleation. We determined how often uveal melanoma (UM) and other intraocular tumors are clinically misdiagnosed among enucleated eyes at the Swedish national ophthalmic pathology laboratory over 28 years, and the lesions responsible.

**Methods:**

In this retrospective, registry-based case series, all enucleation specimens accessioned at the St. Erik Ophthalmic Pathology Laboratory, Stockholm, between 1995 and 2022 under a clinical diagnosis of malignancy were reviewed against pathology reports, referral letters, and clinical records. Predefined criteria identified eyes in which a malignant tumor drove enucleation but was not confirmed histopathologically. In an exploratory analysis, the authors rated the projected visual acuity had enucleation been deferred. The misdiagnosis rate was modeled against calendar year by logistic regression and tested in sensitivity analyses.

**Results:**

Of 24,242 specimens, 1,661 were enucleations, and after predefined exclusions, 1,419 eyes had been enucleated for a presumed tumor. Thirty-nine (2.7%) were misdiagnoses: 21 diagnosed as UM, 11 with another suspected neoplasm, and 7 with a suspected unspecified malignancy. Intraocular hemorrhage was the lesion most often mistaken for UM. Among the 21 μm eyes, the projected visual impact of enucleation was nil in 13 that had no useful vision at enucleation, a loss of useful vision in 1 (decimal acuity 0.25, a benign leiomyoma), and not estimable in 7. The misdiagnosis rate did not change appreciably (odds ratio 0.97 per year, 95% CI 0.94 to 1.01, *P* = 0.16), remained between 2.3% and 4.2% in sensitivity analyses, and did not differ between 1995 and 2010 (2.8%) and 2011–2022 (1.8%; *P* = 0.25). Misdiagnosed eyes were almost always blind, painful, or had opaque media, where clinical and imaging assessment is least reliable.

**Conclusions:**

Clinical misdiagnosis preceded 2.7% of tumor-related enucleations over 28 years, at a stable rate, and intraocular hemorrhage was the most frequent mimic of UM. In an exploratory projection based on incomplete records, misdiagnosis seldom appeared to sacrifice useful vision. The findings reinforce the value of multimodal imaging, ocular-oncology consultation, and consideration of biopsy before enucleation when the diagnosis is uncertain.

## Background

Uveal melanoma (UM) is the most common primary intraocular malignancy in adults, with an incidence of approximately 4 to 7 cases per million per year in populations of predominantly European ancestry [1]. Reported incidence in the Nordic countries is even higher in recent data [2]. Although most tumors are confined to the eye at presentation, UM disseminates hematogenously, and half of patients ultimately die of metastatic disease, predominantly in the liver [3]. The diagnosis typically rests on clinical examination and imaging rather than on tissue biopsy, and most tumors are treated with eye-sparing radiation [4]. An incorrect clinical diagnosis therefore carries two opposite risks: a benign or non-neoplastic lesion may lead to enucleation or radiation, and a melanoma may go untreated when it is mistaken for a benign process.

The accuracy of the clinical diagnosis of UM has improved over several decades. Early series from the Armed Forces Institute of Pathology (AFIP) reported that roughly one in five eyes enucleated for a suspected posterior UM contained a simulating lesion instead [5, 6]. A later AFIP review found an overall misdiagnosis rate of 6.4% and a fall from 12.5% to 1.4% across an 11-year period [7]. The Collaborative Ocular Melanoma Study, with standardized imaging and centralized review, reported a misdiagnosis rate of 0.48% [8]. Because the AFIP series were assembled from globes submitted by general ophthalmologists throughout the United States, they indicate that diagnostic accuracy outside specialized ocular-oncology centers has historically been lower than the 0.48% achieved under study conditions [5–7]. 

Single-center, population-based data on this question are scarce, and the available reports come largely from North America. The only single-center, population-based series in the era of modern imaging reported a 2.0% rate of clinically misdiagnosed posterior UM [9]. In Sweden, intraocular tumor surgery and ophthalmic pathology are centralized: St. Erik Eye Hospital is the national referral center for ocular oncology, and its laboratory is the only ophthalmic pathology service in the country [10]. This structure makes it possible to assess clinical misdiagnosis of UM across an entire national referral population from a single, continuous record. We reviewed all enucleation specimens accessioned over 28 years to determine how often a clinically suspected intraocular malignancy was not confirmed on histopathology, the lesions responsible, and the projected visual consequence of the enucleations. Unlike prior work that also examined the opposite error, namely UM discovered unexpectedly in eyes removed for other indications, we restricted the analysis to eyes in which a malignancy was clinically suspected but not present [11, 12]. 

## Methods

### Setting and data source

St. Erik Eye Hospital, Stockholm, is the national referral center for intraocular tumors in Sweden, and the St. Erik Ophthalmic Pathology Laboratory is the only ophthalmic pathology laboratory in the country. Treatment of a suspected malignant intraocular tumor aims at tumor control and, when compatible with it, at preservation of useful vision, using eye-sparing modalities such as plaque brachytherapy (UM, retinoblastoma), cryotherapy, transpupillary thermotherapy (TTT), and intravitreal or intra-arterial chemotherapy (retinoblastoma). If useful vision cannot be saved, the aim is to preserve the eye; if the eye cannot be preserved, enucleation is indicated. For UM, by far the most common intraocular malignancy treated at the center, enucleation is chosen when the tumor is too large for plaque brachytherapy (largest basal diameter > 16 mm and/or thickness > 10 mm) or unfavorably located for it, for example with circumpapillary growth or extraocular extension exceeding 5 mm in largest diameter. The use of TTT for UM has decreased markedly after it was associated with higher rates of local recurrence and melanoma-related mortality [13]. The threshold for recommending enucleation is lowered for eyes that also have a long-standing total retinal detachment, rubeosis iridis, elevated intraocular pressure that cannot be adequately controlled, or severe discomfort. Because St. Erik Eye Hospital is the only ocular oncology center in Sweden and its laboratory the only ophthalmic pathology service, patients with a clinically suspected intraocular malignancy are, as a rule, referred there for examination regardless of where in the country they live, and specimens with suspected malignancy are sent there regardless of where the surgery is performed. Since 2002, transvitreal incisional biopsy of clinically indeterminate choroidal tumors has been used at the center when clinical examination and imaging do not give the ocular oncologist sufficient confidence in the diagnosis. This date is relevant here because access to intraocular biopsy could plausibly lower the rate of enucleation of misdiagnosed eyes; whether the misdiagnosis rate changed at its introduction was therefore tested (see Statistical Analyses). We searched the laboratory registry for all specimens accessioned between January 1, 1995, and December 31, 2022. Diagnoses were coded with SNOMED [14]. The study followed the tenets of the Declaration of Helsinki and was approved by the Swedish Ethical Review Authority (Reference 2025-05464-01).

### Case selection

Among all accessioned specimens, enucleation specimens were identified. Eyes removed under a registry diagnosis of UM, retinoblastoma, metastasis, or another malignancy were retained for review. One case was excluded because of a misregistered civic identification number. Further exclusions were applied for: tissue misclassified in the registry as a globe (*n* = 7; conjunctiva 3, eyelid 3, unspecified 1); clinically and histopathologically confirmed melanoma in which enucleation was the primary treatment (*n* = 12); clinically and histopathologically confirmed other neoplasia (*n* = 3; basal cell carcinoma 1, sebaceous carcinoma 1, conjunctival carcinoma 1); post-mortem examination of ocular tissue (*n* = 14); and one decompensated phthisical eye with a history of UM previously treated successfully with radiation. Two eyes in which the clinical diagnosis was made within the study period but the enucleation specimen was accessioned in 2023 and 2024 were included as misdiagnoses and, in the time-trend analysis, assigned to the last two years of the study period (2021 and 2022).

### Definition of clinical misdiagnosis

Because access to patient records from referring clinics was limited and digital health records were sparse before 2011, clinical misdiagnosis of UM was defined a priori. “Misdiagnosed” here also includes eyes in which UM was strongly suspected as the primary diagnosis but could not be clinically confirmed. An eye was classified as a misdiagnosed UM when the pathology report did not confirm UM, and UM was not listed solely as a diagnosis of exclusion in the pathology referral but was accompanied by both (a) a clinical presentation compatible with UM and (b) a diagnostic work-up directed at confirming or excluding UM, and either the patient had been clinically diagnosed with UM as the definitive diagnosis in the referral letter or a clinic note, or the clinical findings and work-up strongly supported, without conclusively confirming, UM as the leading diagnosis prompting enucleation of a symptomatic eye.

The same criteria were applied to other intraocular neoplasia, replacing UM with the respective clinically suspected tumor, and to eyes in which an unspecified intraocular malignancy was suspected. The latter group was defined by fulfilling the pathology-report, referral, and clinical-support conditions with “unspecified malignancy” in place of UM.

### Data abstraction and visual-acuity projection

For each misdiagnosed eye we recorded laterality, sex, age, diagnostic work-up, site of clinical assessment, site of enucleation (St. Erik Eye Hospital or an outside provider), and visual acuity. Visual acuity was not an indication for enucleation; it was recorded to estimate what the removal of the eye cost the patient in terms of vision. To gauge whether the misdiagnosis carried a visual cost, each eye was assigned a projected visual-acuity course. Using the histopathologic diagnosis together with the documented preoperative condition of the eye, the authors judged whether the lesion that was actually present, had the eye been retained and managed without enucleation, would have left visual acuity better (+), unchanged (0), or worse (−) than it was at the time of enucleation. An eye was rated unchanged when it was already amaurotic or when the actual lesion offered no realistic prospect of visual gain or further loss, worse when the natural course or the treatment the lesion would have required was expected to reduce acuity, and not estimable when the records did not support a judgment. Each author rated the cases independently, and discrepancies were resolved by discussion. This projection is a structured clinical judgment, not a measured outcome, and it was analyzed as an exploratory secondary outcome.

### Statistical analyses

The denominator for the misdiagnosis rate was the number of eyes enucleated for a presumed tumor, obtained by removing misclassified tissue (*n* = 7), post-mortem specimens (*n* = 14), and eyes that met criteria for neither a diagnosed nor a misdiagnosed malignancy (*n* = 221) from the 1,661 enucleations, leaving 1,419 eyes. To test whether the misdiagnosis rate changed over time, the annual number of misdiagnosed eyes was related to the annual number of enucleated eyes examined at the laboratory by logistic regression, with calendar year entered as a continuous variable. To assess whether the 2002 introduction of transvitreal incisional biopsy altered the rate, a segmented Poisson regression of the annual misdiagnosis count was fitted, with the annual enucleation count as an offset and terms for a level change and a change in trend at 2002. Because classification relied on records that were sparser before 2011, the robustness of the misdiagnosis rate was tested in four sensitivity analyses. We calculated an exact binomial 95% CI for the overall rate; reclassified the 7 eyes enucleated for a suspected unspecified malignancy, the least specific category, as not misdiagnosed; assumed that 5% or 10% of the 221 eyes that met criteria for neither category were unrecognized misdiagnoses, adding them to both numerator and denominator; and compared the rate between 1995 and 2010 and 2011–2022 with Fisher’s exact test, using the annual enucleation totals as denominators. Two-sided *P* values below 0.05 were taken to indicate a time trend or a difference between eras. The remaining analyses were descriptive, with counts and proportions reported without inferential testing.

## Results

**Fig. 1:**
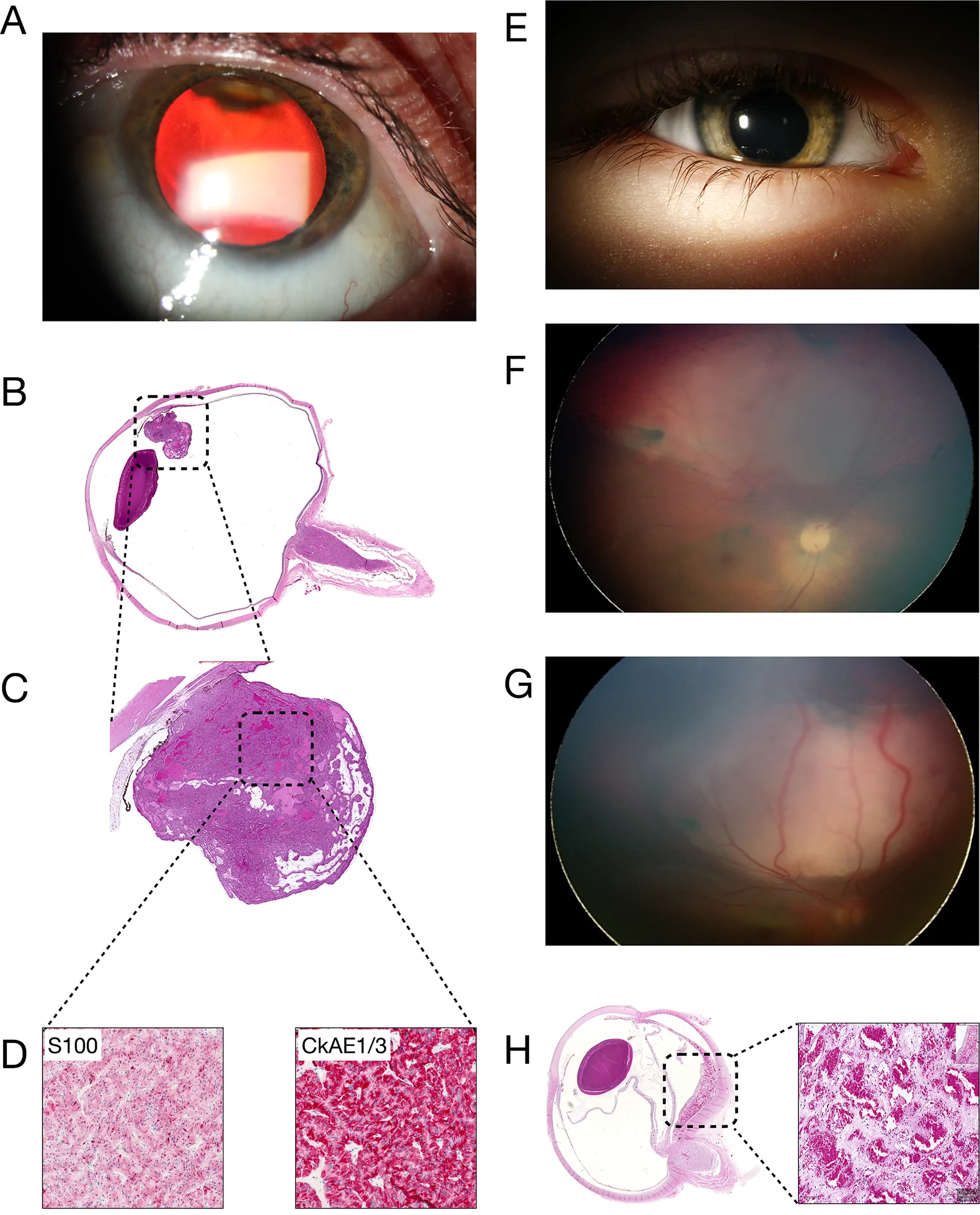
Examples of misdiagnoses. (**A**) A 55-year-old healthy, asymptomatic white man was referred with elevated intraocular pressure (IOP), a sectorial cataract, and a pale tan tumor with dilated vessels in the ciliary body of the left eye. The differential diagnosis included ciliary body medulloepithelioma and, secondarily, ciliary body melanoma. Biopsy was considered unsafe because of the risk of tumor cell seeding, and the eye was enucleated. (**B**) Histopathology showed a nodular endophytic tumor on the inner surface of the ciliary body that appeared to arise from the nonpigmented ciliary epithelium (NPCE). (**C**) The tumor consisted of enlarged, eosinophilic, polygonal cells, with smaller regions of cuboidal and columnar cells, that preserved some morphological characteristics of the NPCE. The nuclei were round to slightly oval, and some contained a small, eccentric nucleolus. The cells grew in trabecular and papillary patterns, with areas of solid and microcystoid growth. Mitoses were rare, < 1 per 10 high-power fields (×40). There were no necrotic areas, rosettes, neural tube-like strands, photoreceptor differentiation, or signs of choroidal invasion. Some strands of epithelial cells rested on prominent periodic acid-Schiff-positive basement membranes. (**D**) Immunohistochemically, the tumor was weakly positive for S100 (Ventana, Oro Valley, AZ, USA) and strongly positive for cytokeratin AE1/AE3 (Thermo Fisher Scientific, Waltham, MA, USA) and vimentin (Ventana, not shown). HMB45 (Ventana, not shown) was negative. A diagnosis of adenoma of the nonpigmented ciliary epithelium (NPCE adenoma) was established. The case has been presented previously [15]. (**E**) In a second example, a then 3-year-old girl with exotropia of the right eye since birth and anisometropia presented with an indeterminate fundus lesion of the right eye. She was otherwise healthy, with no family history of retinoblastoma. Despite successful occlusion therapy (patching), her strabismus recurred, prompting examination by a general ophthalmologist. After the fundus lesion was detected, she was referred for examination under anesthesia. (**F**) A serous detachment and a whitish subretinal mass were found superiorly in the right eye, with surrounding retinal pigment epithelium (RPE) alterations; the left eye was normal. Toxoplasma and Toxocara infection were considered, and after discussion with international colleagues, diffuse choroidal hemangioma and retinoblastoma were raised as possibilities. MRI showed no central nervous system (CNS) lesions. A reddish skin lesion was noted near the right temple, interpreted as a dilated vessel or similar. (**G**) Eighteen months later, the lesion had grown, with total retinal detachment. A repeat orbital MRI raised the possibility of diffuse retinoblastoma, and the eye was enucleated. (**H**) Histopathology showed a diffuse choroidal hemangioma: multiple thin-walled, dilated vessels in the choroid, filling it from the posterior part and extending into the ciliary body, with no increase in mitotic rate. The lesion measured 23 mm in diameter and 1.9 mm in thickness

**Fig. 2:**
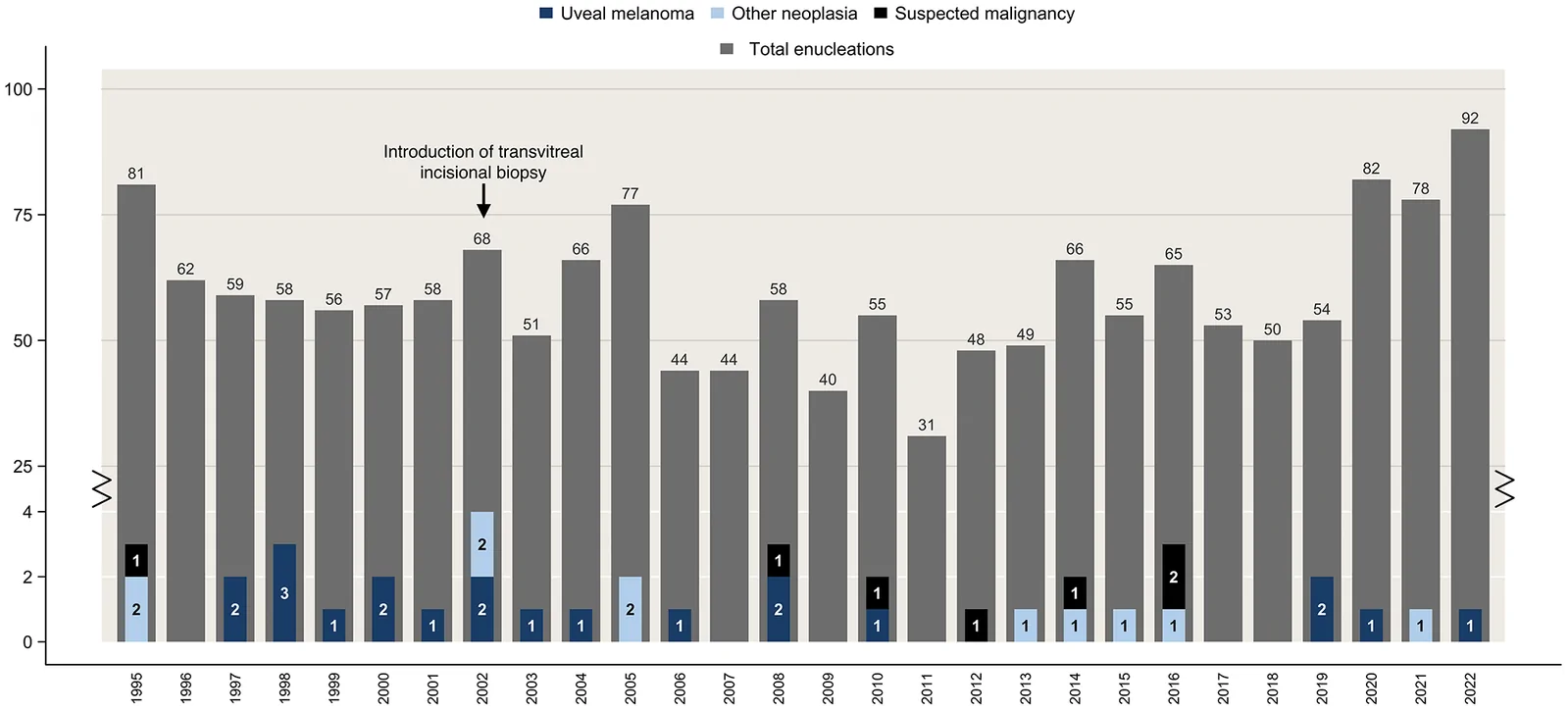
Annual number of enucleated eyes examined at St. Erik Eye Hospital from 1995 through 2022. Gray bars show the total number of enucleations each year. Stacked colored bars show the number of misdiagnosed eyes by category (UM [dark blue], other neoplasia [light blue], and suspected unspecified malignancy [black]). The vertical axis is interrupted between 4 and 25, marked at both ends, so that the small misdiagnosis counts and the larger enucleation totals can be read on one plot. In 2002, transvitreal incisional biopsy for clinically indeterminate choroidal tumors was introduced

Of 24,242 specimens accessioned between 1995 and 2022, 1,661 were enucleations, and 1,367 of these were removed under a diagnosis of UM, retinoblastoma, metastasis, or another malignancy. After the exclusions above, 1,419 eyes had been enucleated for a presumed tumor. Thirty-nine eyes (2.7%) represented a clinical misdiagnosis: 21 clinically diagnosed as UM (Table 1), 11 with another suspected neoplasm (Table 2), and 7 with a suspected unspecified malignancy (Table 3). Two of these cases are shown in Fig. 1: a ciliary body adenoma of the nonpigmented ciliary epithelium that was enucleated under suspicion of medulloepithelioma or melanoma (reported previously [15]), and a diffuse choroidal hemangioma in a young child that was enucleated because a growing, changing lesion raised increasing suspicion of diffuse retinoblastoma. The annual distribution of enucleations and of misdiagnosed eyes is shown in Fig. 2. By excluding misclassified tissue and post-mortem specimens, this proportion is restricted to in vivo enucleations performed for a presumed tumor. It should be read with the understanding that it spans tumors other than UM and eyes assessed and enucleated at providers other than St. Erik.

**Table 1:** Misdiagnosed cases of uveal melanoma in enucleated eyes, 1995–2022 (*n* = 21)

Suspected Dx	Histopathologic Dx	Sex	Age	Side	VA	VA prog.	Out of network	Assessed StE	Enucl. StE	Work-up	Comments
UM	Iris nevus	M	62	L	0	0	Yes	NA	No	PE, ?	Previously amaurotic eye (glaucoma).
UM	Hemorrhage	M	93	R	0	0	Yes	NA	NA	PE, ?	Amaurotic eye with advanced exudative AMD and CNV with hemorrhage.
UM		M	65	L	0	0	Yes	Yes*	No	PE, ?	Previously amaurotic eye. Dark pigment in the anterior chamber angle raised suspicion of UM.
UM	Hemorrhage	F	84	R	NA	NA^	NA	NA	NA	NA	No medical history available. Vitreous hemorrhage.
UM	Traumatic injury	M	77	NA	NA	NA	NA	NA	No	PE, ?	Dementia. Suspected intraocular tumor and pigmented limbal spot noted at diabetes checkup.
UM	Hemorrhage	F	87	L	NA	NA	Yes	No	No	MRI, US, ?	Differential diagnosis CLL recurrence. Radiology suggested UM. Pathology showed subretinal and vitreous hemorrhage.
UM	Hemorrhage	F	85	L	P	0	Yes	Yes	No	B-scan	CNV and subretinal hemorrhage.
UM	Hemorrhage	M	91	L	NA	NA	Yes	No	No	B-scan, TI	CNV and subretinal hemorrhage.
UM	Hemorrhage	F	80	R	P	NA	Yes	Yes	No	PE, B-scan	Ruthenium treatment without tumor regression. Choroidal hematoma was the definitive diagnosis.
UM	Retinal detachment	F	79	R	0	0	Yes	No	No	PE, ?	Amaurotic eye. Onset of pain and a suspicious subretinal lesion prompted enucleation.
UM	Inflammation, unspecified	F	57	R	H	0	Yes	Yes	No	B-scan, MRI	Highly suspicious subretinal lesion on MRI. Report showed nodular scleritis and inflammation.
UM	Hemorrhage	M	85	L	0	0	Yes	No	No	B-scan	Spontaneously perforated eye with a lesion resembling UM on ultrasonography.
UM	Dislocated lens nucleus	F	77	L	NA	NA^	No	No	No	PE, ?	Dislocated lens nucleus mistaken for a UM-like protrusion into the vitreous.
UM	Hemorrhage	M	36	L	0	0	Yes	No	No	PE, B-scan	Amaurotic eye with total glaucoma. Clinical and sonographic suspicion of UM.
UM	Mesectodermal leiomyoma	F	48	L	0.25	-	Yes	Yes	No	PE, B-scan	Ciliary body mesectodermal leiomyoma, 10 × 11 mm.
UM	Hemorrhage	M	94	R	P	0	Yes	Yes	No	PE, Bx, US, MRI	CNV with extensive subretinal hemorrhage. Initial biopsy could not exclude UM.
UM	Retinal vasoproliferative tumor	M	35	L	P	0	Yes	Yes	No	B-scan	Initially correct diagnosis but unresponsive to PDT and cryotherapy; enucleated as suspected UM.
UM	Hemorrhage	M	82	R	P	0	No	Yes	Yes	B-scan	Suprachoroidal hemorrhage.
UM	CNV	F	79	R	P	0	Yes	Yes	No	PE, B-scan	Pathology showed CNV and total retinal detachment.
UM	Hemorrhage	M	78	R	0	0	Yes	Yes	No	PE, B-scan	Juxtachoroidal fibrotic tissue with vascularization.
UM	Fuchs adenoma	M	60	L	NA	NA	Yes	Yes	No	PE, B-scan, ?	Brachytherapy at St. Erik in 2021; enucleation in 2024 showed Fuchs adenoma.

Summarized clinical data for the 21 misdiagnosed cases of uveal melanoma (UM) and their histopathologic diagnoses, 1995–2022. Side: L, left; R, right. Dx, diagnosis. A blank histopathologic diagnosis denotes that no specific lesion was named in the report. Sex: M, male; F, female. Age at enucleation in years. VA, visual acuity at enucleation, graded as 0, no light perception (amaurosis); P, light perception (light perceived without demonstrated projection); H, hand movements; or a decimal value (Snellen decimal acuity). VA prog., projected visual-acuity course, defined as the authors’ estimate of the visual impact of enucleation relative to retaining the eye: 0, no useful vision to lose (no projected change); −, loss of useful vision (a seeing eye removed); ^Likely unfavorable VA prognosis inferred from the pathology report; +, projected gain (not observed in this series); NA, not estimable. Out of network: Yes, patient managed outside St. Erik Eye Hospital (StE). Assessed StE and Enucl. StE: whether the eye was assessed and enucleated at StE, respectively. Work-up, diagnostic methods used. *Assessment based on written correspondence only. ?Work-up information may be incomplete. Abbreviations: AMD, age-related macular degeneration; B-scan, B-scan (brightness-mode) ultrasonography; Bx, biopsy; CLL, chronic lymphocytic leukemia; CNV, choroidal neovascularization; MRI, magnetic resonance imaging; NA, not available; PDT, photodynamic therapy; PE, physical examination; TI, transillumination; US, ultrasonography

**Table 2:** Misdiagnosed cases of other intraocular neoplasia in enucleated eyes, 1995–2022 (*n* = 11)

Suspected Dx	Histopathologic Dx	Sex	Age	Side	VA	VA prog.	Out of network	Assessed StE	Enucl. StE	Work-up	Comments
Retinoblastoma	Inflammation, granulomatous	F	4	R	NA	NA	1	NA	NA	NA	Granulomatous inflammation.
Retinoblastoma	Coats disease	F	1	L	0	0	1	0	0	PE, CT, B-scan	Advanced Coats disease.
Retinoblastoma	Inflammation, granulomatous	F	3	L	0	0	0	1	1	PE, B-scan	Granulomatous inflammation, likely from ocular toxocariasis.
Retinal vasoproliferative tumor	RPE adenoma	F	30	L	NA	0	1	1	1	PE, B-scan	Pathology report showed an RPE adenoma.
Choroidal metastasis	Choroidal hemangioma	F	63	L	0	0	1	0	0	PE, B-scan	Amaurotic eye in a patient with prior breast cancer.
Retinoblastoma	PHPV	F	0	R	0	0	1	1	1	NA	Amaurotic eye in a newborn girl; suspected RB. Persistent hyperplastic primary vitreous.
Choroidal metastasis	Hemorrhage	F	83	R	0	0	1	0	0	PE, CT, ?	Suspected metastasis from prior breast cancer. Hypertonic, amaurotic eye; pathology showed CNV.
Medulloepithelioma	Retinal detachment	M	0	L	0	0	0	1	1	PE, B-scan	Total retinal detachment.
Retinoblastoma	Diffuse choroidal hemangioma°	F	5	R	0	0	1	1	1	PE, US, MRI	Highly suspicious for RB clinically and on MRI.
Retinoblastoma	PHPV°	F	0	L	0	0	1	1	1	PE, US	Highly suspicious for RB clinically and on MRI.
Medulloepithelioma	NPCE adenoma	M	60	L	0.7	-	1	1	1	PE, UBM	Adenoma of the non-pigmented ciliary epithelium. Diagnosed 2022.

Summarized clinical data for the 11 misdiagnosed cases of benign and malignant intraocular neoplasia other than uveal melanoma and their histopathologic diagnoses, 1995–2022. Dx, diagnosis. Sex: M, male; F, female. Side: L, left; R, right. Age in years; 0 denotes age under 1 year. VA, visual acuity at enucleation, graded as 0, no light perception (amaurosis); a decimal value, Snellen decimal acuity; or NA, not available. VA prog., projected visual-acuity course, defined as the authors’ estimate of the visual impact of enucleation relative to retaining the eye: 0, no useful vision to lose (no projected change); −, loss of useful vision (a seeing eye removed); NA, not estimable. Out of network, Assessed at St. Erik Eye Hospital (StE), and Enucl. StE: 1, yes; 0, no. Out of network denotes patients managed outside StE; Assessed StE and Enucl. StE denote whether the eye was assessed and enucleated at StE, respectively. Work-up, diagnostic methods used. ?Work-up information may be incomplete. °Diagnosis offered as a less likely differential. Abbreviations: B-scan, B-scan (brightness-mode) ultrasonography; CNV, choroidal neovascularization; CT, computed tomography; MRI, magnetic resonance imaging; NA, not available; NPCE, non-pigmented ciliary epithelium; PE, physical examination; PHPV, persistent hyperplastic primary vitreous; RB, retinoblastoma; RPE, retinal pigment epithelium; UBM, ultrasound biomicroscopy; US, ultrasonography (unspecified modality)

**Table 3:** Cases of suspected unspecified intraocular tumors in enucleated eyes, 1995–2022 (*n* = 7)

Suspected Dx	Histopathologic Dx	Sex	Age	Side	VA	VA prog.	Out of network	Assessed StE	Enucl. StE	Work-up	Comments
Malignant tumor		M	58	L	0	0	1	0	0	PE, CT, ?	Amaurotic eye with high suspicion of malignancy.
Malignant tumor	Hemorrhage	F	89	R	0	0	1	NA	0	NA	Painful eye with extensive choroidal detachment and suspected malignancy.
Malignant tumor	Hemorrhage	F	78	R	0	0	1	1	0	PE, B-scan, MRI	Amaurotic eye. Prior hysterectomy. MRI unremarkable. Systemic symptoms unclear.
Malignant tumor	Hemorrhage	F	88	NA	NA	NA^	1	0	0	PE, Bx, ?	Suspected subretinal tumor. Pathology report showed total retinal detachment.
Malignant tumor	Retinal hemangioma	F	16	R	0.1	0	1	1	0	PE, MRI, ?	Known hemangioma unresponsive to treatment; retinal detachment raised suspicion of malignancy.
Malignant tumor	Necrosis	M	86	L	NA	0	1	0	0	PE, MRI, ?	Swollen, hemorrhagic eye suspicious for malignancy.
Malignant tumor	Degeneration	M	32	L	0	0	1	0	0	NA	Total retinal detachment and secondary glaucoma suspicious for malignancy. Systemic symptoms unclear.

Summarized clinical data for the 7 eyes enucleated for a suspected but unspecified intraocular malignancy and their histopathologic diagnoses, 1995–2022. Dx, diagnosis. A blank histopathologic diagnosis denotes that no specific lesion was named in the report. Sex: M, male; F, female. Age at enucleation in years. Side: L, left; R, right. VA, visual acuity at enucleation, graded as 0, no light perception (amaurosis); a decimal value, Snellen decimal acuity; or NA, not available. VA prog., projected visual-acuity course, defined as the authors’ estimate of the visual impact of enucleation relative to retaining the eye: 0, no useful vision to lose (no projected change); NA, not estimable. Out of network, Assessed St. Erik Eye Hospital (StE), and Enucl. StE: 1, yes; 0, no. Out of network denotes patients managed outside StE; Assessed StE and Enucl. StE denote whether the eye was assessed and enucleated at StE, respectively. Work-up, diagnostic methods used

?Work-up information may be incomplete. ^Likely unfavorable VA prognosis inferred from the pathology report. Abbreviations: B-scan, B-scan (brightness-mode) ultrasonography; Bx, biopsy; CT, computed tomography; MRI, magnetic resonance imaging; NA, not available; PE, physical examination

### Misdiagnosed uveal melanoma

**Fig. 3:**
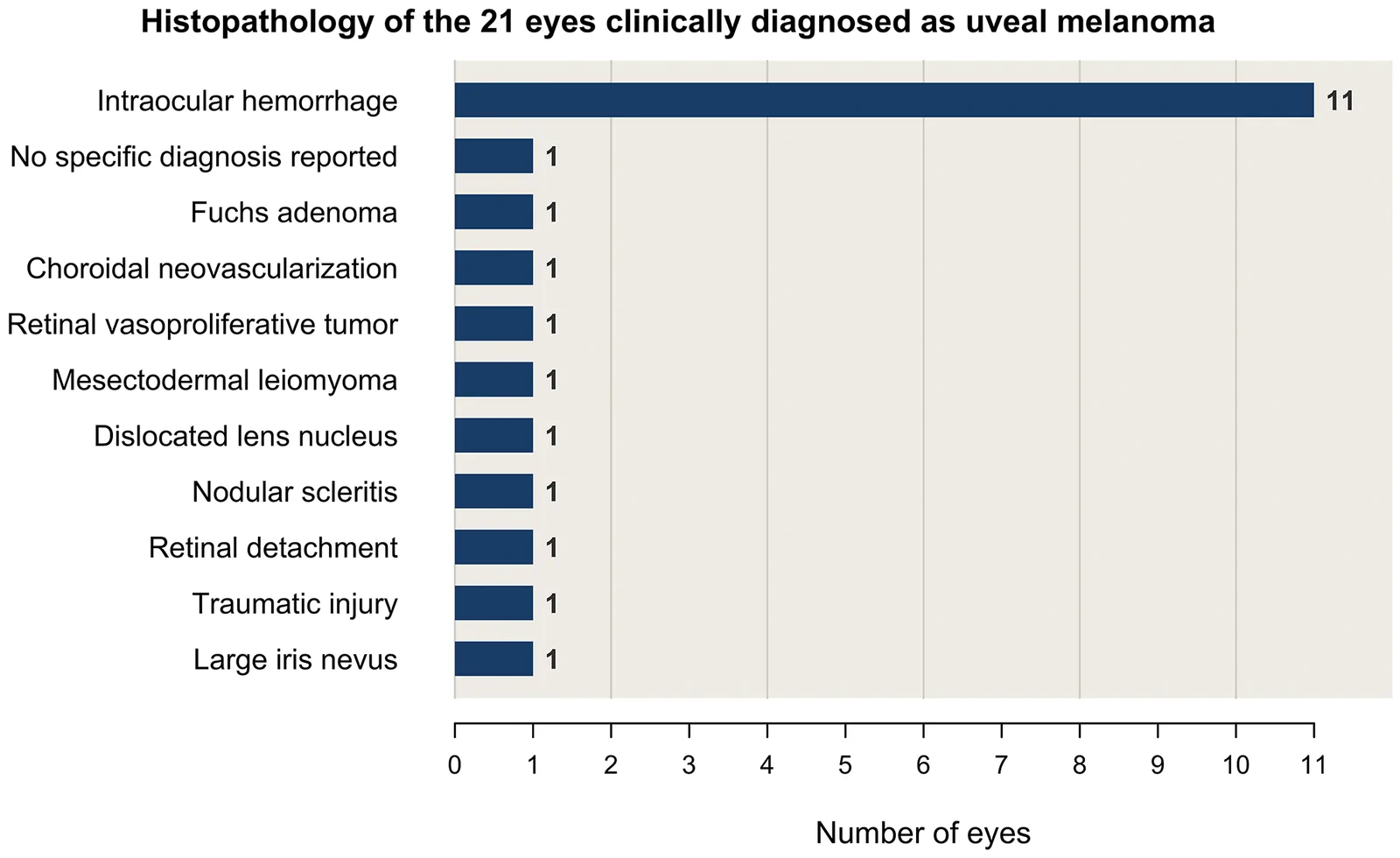
Histopathologic diagnoses of the 21 eyes clinically diagnosed as, or strongly suspected of harboring, uveal melanoma. Intraocular hemorrhage was the most frequent finding

Twenty-one eyes were clinically diagnosed as, or strongly suspected of harboring, UM but contained another lesion (Table 1; Fig. 3). The most frequent histopathologic finding was intraocular hemorrhage (11 of 21 eyes, 52%), including subretinal hemorrhage associated with choroidal neovascularization, suprachoroidal hemorrhage, and choroidal hematoma after ruthenium plaque treatment. The remaining diagnoses were single instances of choroidal neovascular membrane, retinal detachment, nodular scleritis with unspecified inflammation, iris nevus, ciliary body mesectodermal leiomyoma, retinal vasoproliferative tumor, dislocated lens nucleus protruding into the vitreous, traumatic injury, Fuchs adenoma, and one eye in which no neoplasm was specified. Patients were 12 men (57%) and 9 women (43%), with a mean age of 73 years (SD 17) and a median of 79 years (IQR 62 to 85, range 35 to 94). Visual acuity at enucleation was a decimal acuity of 0.25 in 1 eye (Snellen 20/80, logMAR 0.60), light perception or hand movements in 7 eyes, and no light perception in 7; it was not available in the remaining 6. Fundus visibility was not a systematically recorded item, so the exact number of eyes in which the lesion could not be visualized cannot be stated. The documented findings nevertheless indicate that direct visualization was precluded or unreliable in most eyes: 14 of the 21 had no light perception or light perception to hand movements only, and the records describe vitreous hemorrhage, opaque media, or a perforated globe in several (Table 1).

### Misdiagnosed other neoplasia

Eleven eyes were enucleated under a clinical diagnosis of an intraocular neoplasm other than UM (Table 2). Retinoblastoma was the suspected diagnosis in six eyes (pseudoretinoblastoma), simulated by granulomatous inflammation (2 eyes, one attributed to ocular toxocariasis), Coats disease, persistent hyperplastic primary vitreous (2 eyes), and diffuse choroidal hemangioma. Suspected choroidal metastasis in two patients with prior breast cancer proved to be choroidal hemangioma and choroidal neovascularization. The remaining eyes comprised a suspected retinal vasoproliferative tumor that was an adenoma of the retinal pigment epithelium, and two suspected medulloepitheliomas that were a retinal detachment and an adenoma of the non-pigmented ciliary epithelium. Seven of the 11 patients were children.

### Suspected unspecified malignancy

Seven eyes were enucleated for a suspected intraocular malignancy that was not further specified clinically (Table 3). Histopathology showed intraocular hemorrhage in three eyes and, in single eyes, retinal hemangioma, necrosis, and degeneration, with one eye in which no neoplasm was specified. Most were amaurotic or painful eyes with opaque or hemorrhagic media.

### Time trend of misdiagnosis

The annual number of misdiagnosed eyes ranged from 0 to 4 (Fig. 2). In a logistic regression with calendar year as a continuous variable, the annual odds of misdiagnosis per enucleated eye did not change (odds ratio 0.97 per year, 95% CI 0.94 to 1.01, *P* = 0.16; odds ratio 0.77 per decade, 95% CI 0.52 to 1.11), and the rate was stable across the 28 years. In a segmented Poisson regression of the annual misdiagnosis count (annual enucleations as offset), with terms for a level change and a change in trend at the 2002 introduction of transvitreal incisional biopsy, neither indicated an effect: the rate did not step at introduction (rate ratio 1.25, 95% CI 0.29 to 5.32, *P* = 0.76), and the subsequent trend did not change (rate ratio 0.97 per year, 95% CI 0.73 to 1.29, *P* = 0.85).

### Sensitivity analyses

The overall misdiagnosis rate was 39 of 1,419 eyes (2.7%, 95% CI 2.0 to 3.7). Under the strict definition, with the 7 eyes enucleated for a suspected unspecified malignancy reclassified as not misdiagnosed, the rate was 32 of 1,412 (2.3%, 95% CI 1.6 to 3.2). If 5% or 10% of the 221 eyes that met criteria for neither category had been unrecognized misdiagnoses, the rate would have been 3.5% (95% CI 2.6 to 4.6) or 4.2% (95% CI 3.3 to 5.4). The rate did not differ between the sparse-record era, 1995–2010 (26 of 934, 2.8%, 95% CI 1.8 to 4.1), and the digital-record era, 2011–2022 (13 of 723, 1.8%, 95% CI 1.0 to 3.1; odds ratio 1.56, 95% CI 0.77 to 3.34, *P* = 0.25). The misdiagnosis rate thus remained between approximately 2% and 4% under all scenarios.

### Projected visual-acuity course

In an exploratory analysis, visual-acuity course was projected from the histopathologic diagnosis together with the documented preoperative status of each eye, rather than measured, to estimate whether enucleation had cost useful vision. Visual acuity at enucleation was unavailable in 10 of the 39 eyes (26%), and the projected course was not estimable in 9. Among the 21 eyes misdiagnosed as UM, the projected visual impact of enucleation was nil in 13 eyes, which had no useful vision at enucleation (most were amaurotic, for example from longstanding total retinal detachment); not estimable in 7 because of missing data; and a loss of useful vision in 1 eye. That single eye was the only one of the 21 with measurable vision at enucleation (decimal acuity 0.25, Snellen 20/80) and contained a benign ciliary body mesectodermal leiomyoma, so its removal did sacrifice a seeing eye, and a correct diagnosis might have allowed an attempt to preserve vision. In the remaining 20 eyes, enucleation entailed no known loss of useful vision because these eyes were already non-seeing or could not be assessed. Among the 18 eyes in the other two groups (Tables 2 and 3), the projected impact was nil in 15, not estimable in 2, and a loss of useful vision in 1: the eye with an adenoma of the non-pigmented ciliary epithelium and a decimal acuity of 0.7. No eye in any group was projected to gain vision had enucleation been deferred. Referrals that did not meet the misdiagnosis definition, and were therefore not counted here, included blind painful eyes and perforated phthisical eyes after trauma, infection, or glaucoma.

## Discussion

Across 28 years of nationwide referral-based data, a clinically suspected intraocular malignancy was not confirmed on histopathology in 2.7% of eyes enucleated for a presumed tumor. For UM specifically, 21 eyes were misdiagnosed. This proportion sits between the 0.48% reported under the standardized conditions of the Collaborative Ocular Melanoma Study and the substantially higher rates from the older AFIP series drawn largely from general practice [5–8]. By coincidence, our rate was identical to that of an earlier Mayo Clinic series, in which 6 of 224 eyes (2.7%) enucleated for a clinically suspected melanoma proved to be mimicking lesions [16]. Because our data derive from the single laboratory serving the national referral pathway, the figure reflects routine practice across a whole country rather than the performance of a selected referral cohort.

Intraocular hemorrhage was the lesion most often mistaken for UM and accounted for 15 of the 39 misdiagnosed eyes overall (38%), a pattern consistent with historical series in which hemorrhagic and detachment-related lesions dominated the simulating diagnoses [5, 6]. Hemorrhage and necrosis are difficult to distinguish from a pigmented tumor when the media are opaque, and necrotic tumors can themselves mimic inflammation; both directions of error have been described [11]. In our series the misdiagnosed eyes were typically amaurotic, painful, or had opaque media, the same circumstances in which clinical and imaging assessment is least reliable. Several of the hemorrhagic lesions in this series arose from choroidal neovascularization in exudative age-related macular degeneration, and peripheral exudative hemorrhagic chorioretinopathy, the peripheral end of the polypoidal choroidal vasculopathy spectrum, led to referral for suspected choroidal melanoma in 173 eyes in one series [17]. Ultrasonography retains its value precisely when the media are opaque, and it can separate these lesions: hemorrhage lacks internal blood flow, whereas melanoma is vascularized, and in an early color Doppler series, internal flow was demonstrated in 39 neoplastic lesions but in none of three tumor-simulating lesions [18]. Hemorrhage is also expected to regress on short-interval re-examination, whereas melanoma is not. We therefore suggest that an eye with opaque media and a presumed intraocular mass undergo B-scan ultrasonography with color Doppler where available, short-interval re-imaging, and review by an ocular oncology service before enucleation is decided.

Six of the 11 non-UM neoplasia misdiagnoses occurred in children with suspected retinoblastoma, a situation known as pseudoretinoblastoma. Coats disease, persistent hyperplastic primary vitreous, granulomatous inflammation, and diffuse choroidal hemangioma accounted for the misdiagnoses in children with suspected retinoblastoma here, matching the simulating lesions that dominate larger series [19]. The reported frequency of pseudoretinoblastoma depends heavily on the setting: 22% of 2,775 patients referred for suspected retinoblastoma in the United States, 31% of 607 referrals in Turkey, 1.4% of 280 eyes enucleated for presumed retinoblastoma in India, and 12.9% of 70 enucleated eyes in China [19–22]. Our six cases in 28 years are consistent with a low frequency at the enucleation stage in a centralized national system, although the number of eyes enucleated for suspected retinoblastoma was not available as a denominator. Unlike in adults, diagnostic biopsy offers no way out of this uncertainty: biopsy of a suspected retinoblastoma is generally contraindicated because of the risk of extraocular tumor seeding, so the diagnosis rests on examination under anesthesia and imaging, and enucleation of an occasional simulating lesion is difficult to eliminate entirely [23]. Among adults, ciliary body lesions, including mesectodermal leiomyoma and adenoma of the non-pigmented ciliary epithelium, and an adenoma of the retinal pigment epithelium were each mistaken for a malignant tumor, reflecting the difficulty of characterizing solid lesions of the ciliary body and peripheral fundus.

The exploratory visual-acuity projection suggests that misdiagnosis in this setting seldom cost useful vision: no eye was projected to gain vision had enucleation been deferred, and most were already amaurotic. This contrasts with the graver opposite error, an unrecognized melanoma left untreated, which could shorten survival [11, 24–27]. We did not measure that error, because eyes containing an unsuspected UM removed for an unrelated indication fell outside our case definition; however, prior work indicates that such cases still occur and are likely underreported [11, 12]. The practical implication is that when the media are opaque, imaging such as B-scan ultrasonography or magnetic resonance imaging, review by an experienced ocular oncologist, and biopsy where suitable, should precede enucleation.

Diagnostic intraocular biopsy is a further option for the uncertain case. When clinical and imaging features are insufficiently distinct, transvitreal or transretinal incisional biopsy and fine-needle aspiration can confirm or exclude malignancy with high sensitivity and specificity [28–30]. In a series of clinically indeterminate choroidal tumors at our institution, incisional biopsy yielded adequate tissue in 95% of cases and detected malignancy with a sensitivity of 0.97 and a specificity of 1.00, and transvitreal biopsy provides tissue for both diagnosis and prognostic testing [28, 31]. When the eye retains useful vision and the diagnosis is in doubt, biopsy offers a direct way to avoid enucleating a benign lesion. However, biopsy is not performed in all centers, it is not free of risk, and it is least feasible in the blind, painful, or media-opaque eyes that made up much of the present cohort, where B-scan ultrasonography, magnetic resonance imaging, and ocular-oncology review remain the practical safeguards. When findings remain equivocal after full imaging, we suggest multidisciplinary review at an ocular oncology center and biopsy whenever technically feasible, with the caveats that most UM can be diagnosed accurately without tissue confirmation, that biopsy is contraindicated in suspected retinoblastoma, and that when a biopsy confirms malignancy, definitive treatment follows within a few weeks, during which clinically evident tumor growth is uncommon [8, 23]. 

The 2002 introduction of biopsy was not followed by a detectable change in the rate of enucleation for a misdiagnosed tumor. This finding is expected rather than informative, however, because the rate counts only misdiagnoses among eyes that were still enucleated, not the indeterminate eyes that biopsy spared from enucleation. A plausible further reason is that biopsy and misdiagnosis act on different eyes. Biopsy is offered for lesions that already appear indeterminate, where uncertainty prompts additional imaging, multidisciplinary discussion, and tissue sampling. The misdiagnosed eyes were largely the opposite: blind eyes with opaque media in which a tumor was assumed, or lesions read with confidence as melanoma. Diagnostic error therefore arose mainly where suspicion was never raised, so a procedure reserved for the doubtful case would not be expected to lower it. The persistence of intraocular hemorrhage as the most frequent mimic throughout the period fits this interpretation, since hemorrhage in a blind, media-opaque eye is often taken for melanoma without prompting the work-up that leads to biopsy. Improved imaging over the period might likewise have been expected to reduce misdiagnosis, but any effect may be limited for the same reason: imaging helps most when a lesion is recognized as uncertain and fully worked up, and least in the opaque or confidently diagnosed eyes where these errors occurred.

### Strengths and limitations

The main strength is a continuous 28-year record from the only ophthalmic pathology laboratory in the country, with case-level review of every misdiagnosed eye rather than a coded summary. Limitations follow from the retrospective design. Records from referring clinics were incomplete, and digital records before 2011 were sparse, so classification relied in part on referral letters. Sparse records could hide misdiagnoses, and well-documented work-ups could favor classification as misdiagnosis; the sensitivity analyses address both possibilities and indicate that the rate remained between approximately 2% and 4% under alternative classification assumptions. Histopathology was not centrally re-reviewed for this study. The distinction between a malignant tumor and its simulating lesions is usually clear on light microscopy, and all reports were issued by subspecialized ophthalmic pathologists at a single laboratory, but exceptions exist and interpretation differences cannot be excluded. In addition, not all tissue is embedded in paraffin: despite careful gross examination and documentation, a minute tumor could in principle escape the histologic sections. Both mechanisms are expected to affect few cases, a degree of misclassification covered by the sensitivity analyses.

The denominator depends on the exclusion logic described in the Methods, and the misdiagnosis proportion combines UM with other tumors and includes eyes assessed and enucleated outside St. Erik. Submission of enucleation specimens to our laboratory is customary rather than mandated, so an enucleated eye examined at a general pathology department, or not submitted for pathology at all, would have escaped the registry. Such losses are, however, likely very rare. Swedish health care is a single-payer system with consensus-based referral routes that are similar throughout the country, the laboratory has been the only ophthalmic pathology service in Sweden since the 1960s, and enucleated eyes are sent to it as a rule, with very few exceptions. When malignancy is suspected, submission is virtually universal, so the eyes at the center of this study should be nearly completely captured, even though the denominator and numerator cannot be guaranteed to be complete.

For the time-trend analysis the denominator was the annual number of enucleations rather than the annual number of tumor enucleations, which was not available by year; this makes the per-year denominator broader than the one used for the overall 2.7% rate. We did not capture eyes containing an unsuspected malignancy removed for another indication, so the analysis addresses only one direction of diagnostic error. Finally, the visual-acuity projection is a subjective estimate rather than a measured outcome and rests on the same incomplete records: visual acuity at enucleation was unavailable in 10 of the 39 eyes (26%), the projected course was not estimable in 9, and fundus visibility was not systematically recorded. This analysis is therefore exploratory, and its results should be interpreted with caution.

## Conclusions

In a nationwide referral-based record spanning 28 years, clinical misdiagnosis preceded 2.7% of tumor-related enucleations, with intraocular hemorrhage the most frequent mimic of UM, and the rate was stable over the period and across sensitivity analyses. Misdiagnosis occurred almost exclusively in blind, painful, or media-opaque eyes and, in an exploratory projection based on incomplete records, rarely entailed loss of useful vision. The findings reaffirm the value of multimodal imaging, including color Doppler ultrasonography when the media are opaque, ocular-oncology consultation, and intraocular biopsy where feasible, before enucleation when the lesion cannot be directly visualized.

## Data Availability

The datasets generated and analyzed during the current study are not publicly available because they contain potentially identifying patient information and are subject to restrictions under Swedish law and the conditions of the ethical approval. De-identified data are available from the corresponding author on reasonable request, subject to the necessary permissions.

## Abbreviations

AFIP: Armed Forces Institute of Pathology
AMD: Age-related macular degeneration
B-scan: Brightness-mode ultrasonography
Bx: Biopsy
CI: Confidence interval
CLL: Chronic lymphocytic leukemia
CNS: Central nervous system
CNV: Choroidal neovascularization
CT: Computed tomography
Dx: Diagnosis
IOP: Intraocular pressure
IQR: Interquartile range
MRI: Magnetic resonance imaging
NA: Not available
NPCE: Nonpigmented ciliary epithelium
OR: Odds ratio
PDT: Photodynamic therapy
PE: Physical examination
PHPV: Persistent hyperplastic primary vitreous
RB: Retinoblastoma
RPE: Retinal pigment epithelium
SD: Standard deviation
SNOMED: Systematized Nomenclature of Medicine
TI: Transillumination
TTT: Transpupillary thermotherapy
UBM: Ultrasound biomicroscopy
UM: Uveal melanoma
US: Ultrasonography
VA: Visual acuity
